# Influence of nitrate supplementation on motor unit activity during recovery following a sustained ischemic contraction in recreationally active young males

**DOI:** 10.1007/s00394-024-03440-9

**Published:** 2024-05-29

**Authors:** Ozcan Esen, Stephen J. Bailey, Daniel W. Stashuk, Glyn Howatson, Stuart Goodall

**Affiliations:** 1 Department of Sport, Exercise and Rehabilitation, Northumbria University, Newcastle-Upon-Tyne, NE1 8ST UK; 2 School of Sport, Exercise and Health Sciences, Loughborough University, Loughborough, UK; 3 Department of Health Professions, Manchester Metropolitan University, Manchester, UK; 4 Department of Systems Design Engineering, University of Waterloo, Waterloo, ON Canada; 5 Water Research Group, North West University, Potchefstroom, South Africa; 6 Physical Activity, Sport and Recreation Research Focus Area, Faculty of Health Sciences, North-West University, Potchefstroom, South Africa

**Keywords:** Beetroot juice, Electromyography, Motor unit, Nitric oxide, Recovery

## Abstract

**Purpose:**

Dietary nitrate (NO_3_^−^) supplementation enhances muscle blood flow and metabolic efficiency in hypoxia, however, its efficacy on neuromuscular function and specifically, the effect on motor unit (MU) activity is less clear. We investigated whether NO_3_^−^ supplementation affected MU activity following a 3 min sustained ischemic contraction and whether this is influenced by blood flow restriction (BFR) during the recovery period.

**Method:**

In a randomized, double-blinded, cross-over design, 14 males (mean ± SD, 25 ± 6 years) completed two trials following 5 days of supplementation with NO_3_^−^-rich (NIT) or NO_3_^−^-depleted (PLA) beetroot juice to modify plasma nitrite (NO_2_^−^) concentration (482 ± 92 vs. 198 ± 48 nmol·L^−1^, *p* < 0.001). Intramuscular electromyography was used to assess MU potential (MUP) size (duration and area) and mean firing rates (MUFR) during a 3 min submaximal (25% MVC) isometric contraction with BFR. These variables were also assessed during a 90 s recovery period with the first half completed with, and the second half completed without, BFR.

**Results:**

The change in MUP area and MUFR, did not differ between conditions (all p > 0.05), but NIT elicited a reduction in MUP recovery time during brief isometric contractions (*p* < 0.001), and during recoveries with (*p* = 0.002) and without (*p* = 0.012) BFR.

**Conclusion:**

These novel observations improve understanding of the effects of NO_3_^−^ on the recovery of neuromuscular function post-exercise and might have implications for recovery of muscle contractile function.

**Trial registration:**

The study was registered on clinicaltrials.gov with ID of NCT05993715 on August 08, 2023.

## Introduction

Dietary nitrate (NO_3_^−^) is a nitric oxide (NO) precursor, particularly under acidic and hypoxic conditions [1], with the potential to enhance skeletal muscle vasodilation, metabolism, and contractility [1, 2]. NO_3_^−^ supplementation has been reported to enhance muscle blood flow [3], and metabolic efficiency [4, 5] in hypoxia. However, the efficacy of NO_3_^−^ supplementation to enhance neuromuscular function [6–9] and its specific effect on motor unit (MU) activity [10, 11] is less clear having received limited empirical investigation, particularly in hypoxic conditions. Increasing NO has been reported to facilitate neurotransmitter (e.g., acetylcholine) release at the neuromuscular junction (NMJ) [12–14], via post-translational S-nitrosylation of key regulatory protein thiols [15], which could underpin any impact of NO_3_^−^ supplementation on MU activity during skeletal muscle contraction.

Principally, NO_3_^−^ supplementation has been evaluated for its potential to modulate physiological responses during exercise [2]. While previous data show some promise for beneficial effects of NO_3_^−^ supplementation on recovery of muscle function (i.e. attenuation in decrements in countermovement jumps) [16, 17], the potential impact of NO_3_^−^ supplementation on neuromuscular level recovery following exercise is unknown. From a metabolic standpoint, there is evidence that NO_3_^−^ supplementation reduces accumulation of inorganic phosphate (Pi) and phosphocreatine (PCr) degradation during, and accelerates PCr recovery kinetics following, knee extensor exercise completed in normobaric hypoxia [4, 18]. Faster PCr recovery in hypoxia has been attributed to increased BF and local perfusion due to enhanced NO production after NO_3_^−^ supplementation [18]. While these findings suggested that NO_3_^−^ supplementation was effective in improving post-exercise metabolic recovery in hypoxia, the effect of NO_3_^−^ supplementation on recovery of MU activity following muscular work completed when O_2_ delivery is impaired has not been determined previously. Since the muscle metabolic milieu can alter neural drive [19], NO_3_^−^ supplementation could impact MU activity and NMJ function by lowering intramuscular metabolic perturbation (e.g., attenuated accumulation of inorganic Pi) [4]. Further, given that metabolic homeostasis can impact cross-bridge cycling and neuromuscular transmission, and since NO has been reported to augment depolarisation of muscle fibres by increasing acetylcholine activity [20], NO_3_^−^ supplementation has the potential to expedite the restoration of MU activity after prolonged muscle contractile activity. It is also known that, during a sustained submaximal isometric skeletal muscle contraction completed under ischemia, MU firing rates (MUFRs) decrease and do not recover post contraction if ischemia is maintained [21].

Altered MUFR during ischemia might be partly due to increased activation of type IV inhibitory afferents in response to metabolite accumulation, with this effect rapidly receding once BF is restored [22, 23]. Indeed, this contention is supported by a recent study concluding that changes in BF positively correlate with changes in MUFR [24]. Interestingly, NO_3_^−^ supplementation (~ 8.2 mmol/day) has been reported to result in a greater hyperaemia following a bout (5 min) of whole limb ischemia in the quadriceps muscle [25]. Therefore, since NO_3_^−^ supplementation has the potential to enhance post contraction hyperaemia, and thereby expedite restoration of muscle metabolic homeostasis, this could facilitate faster restoration of MUFR after a sustained ischemic muscle contraction after NO_3_^−^ supplementation. However, no study has evaluated changes of MUFR during recovery after an ischemic, isometric muscle contraction following NO_3_^−^ supplementation.

The aim of this study was to determine whether NO_3_^−^ supplementation modulates MU activity following a sustained ischemic contraction and whether any such effects differ with and without blood flow restriction (BFR). It was hypothesised that there would be a reduction in the MUFR throughout a sustained contraction and an increase once ischemia ceased, and that NO_3_^−^ supplementation would expedite this recovery of MUFR. It was also hypothesized that there would be increase in MU characteristics (MU potential [MUP] duration and area) throughout the sustained contraction, and these would decrease when ischemia ended, and that NO_3_^−^ supplementation would expedite the decrease in MU characteristics.

## Methodology

### Participants

The sample size of this study was based on a priori calculation using G*Power software (version 3.1.9.4, Universität, Düsseldorf, Germany). Based on a study by Husmann et al. [26] who determined the effects of beetroot juice vs placebo supplement on muscle contraction performance a total sample of 14 participants was required. The sample was based on a medium standardized effect size of 0.75. A f-test family was used with repeated measures within-between interaction, a power of 0.8, and alpha set at 0.05. Fourteen healthy, recreationally active, young males (mean ± SD age 24 ± 6 years, body mass 70.2 ± 11.9 kg, stature 174 ± 10 cm) volunteered for this study. Participants were regularly involved in multiple sports/forms of activity with an average activity level of 6 ± 3 h per week. The protocols, risks, and benefits of participating were explained before obtaining written informed consent. This study was approved by the Manchester Metropolitan University Research Ethics Committee in accordance with the Declaration of Helsinki (reference no: 5951).

### Experimental design

Participants visited the laboratory for one familiarisation session and two experimental sessions on two separate occasions at a similar time of day (± 2 h). On one occasion, participants underwent NO_3_^−^ supplementation (NIT) with a placebo (PLA) consumed on the other laboratory visit. The interventions were applied in a randomized, cross-over, double-blind design. Randomization and blinding were designed by an independent researcher who had no further involvement in the present study. The randomization and blinding were held until the end of the study. The two conditions were separated by 7 ± 1 days to ensure plasma NO_2_^−^ concentration returned to baseline [27]. Participants were asked to maintain habitual physical activity and refrain from strenuous activity 24 h prior to each trial. Participants were also asked to record a 7-day physical activity diary and a 3-day dietary intake before the first trial, which was repeated prior to the second. Participants were also requested to abstain from alcohol, caffeine, and nutritional supplements 24 h prior to the trial day, and to not use antibacterial mouthwash throughout the experimental period.

**Fig. 1:**
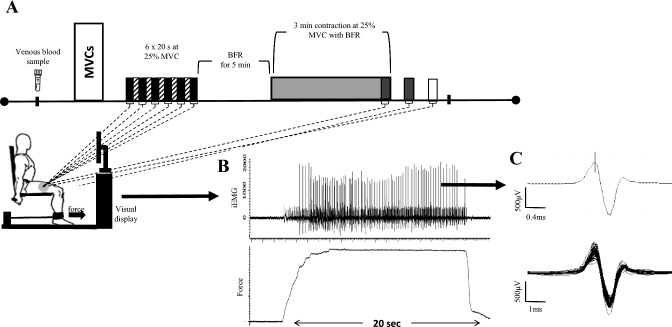
Experimental procedure and a representative intramuscular electromyographic signal (iEMG). **A** Schematic of the dynamometer, muscle contraction procedure, blood sample measurements. **B** An iEMG signal (above) and force tracing (below) from a participant during a sustained isometric contraction at 25% MVC. **C** A single MUP extracted from the iEMG signal shown in **B** and several overlaid MUPs (shimmer plot) extracted across multiple MU firings, respectively. Black boxes: brief muscle contractions; boxes with gradient fill: rests between 6 × 20 s contractions; dark grey boxes: contractions with blood flow restriction (BFR); white box: contraction after releasing BFR

Before the supplementation trials, participants conducted a familiarisation session which included multiple contractions lasting 12–15 s at 25% MVC with and without BFR. Following the completion of this initial familiarisation, in each experimental trial, participants performed an identical protocol of isometric voluntary contractions performed with the dominant knee extensors following the collection of a blood sample (Fig. 1). Briefly, following completion of maximum voluntary contractions (MVCs), a target intensity of 25% MVC was displayed on a monitor in front of the participants to provide force feedback. The rest of the protocol involved, in sequence, 6 × 20 s submaximal (25% MVC) isometric contractions, 8 min of BFR with a sustained isometric contraction at 25% MVC during the final 3 min of the BFR time. Once the 3 min contraction was completed, a 45 s rest period began and, with BFR maintained, participants performed a 20 s, 25% MVC contraction (recovery 1). The BFR was then released, and participants had another 45 s rest before performing a final 20 s 25% MVC contraction (recovery 2). The duration of contraction (20 s) was based on previous work which utilized ranges between 15 to 20 s during iEMG data collection [28, 29]. Such a timeframe is sufficient and appropriate for iEMG data acquisitions as more 10 s of contraction are required to achieve 20 or more appropriate MUP trains [30]. Intramuscular electromyography (iEMG) was recorded from the *m.vastus lateralis* (VL) during all muscle contractions, except during MVCs.

### Supplementation and strength assessment

Participants ingested 2 × 70 mL/day shots of concentrate NO_3_^—^rich (NIT: ~ 12.8 mmol/day NO_3_^−^) or NO_3_^−^—depleted (PLA: ~ 0.08 mmol/day NO_3_^−^) beetroot juice (both supplied by Beet It, James White Drinks Ltd., Ipswich, UK). The NO_3_^−^—depleted beetroot juice was generated using a standard ion exchange resin, as described previously [31]. Two shots were supplemented for 5 days; one each morning (~ 9 am) and one each evening (~ 9 pm) except for the day of the experimental trial when both shots were taken together 2.5 h before the experimental trial [27, 32].

### Force assessment

Participants sat in an isometric knee-extensor strength testing chair with hip and knees flexed at 90°. The chest and waist strapping secured participants tightly to the chair, minimizing upper body movements. A custom-built force transducer was adjusted around the leg being tested [33], 30 cm below the centre of the knee joint. Following familiarization and warm up with a series of submaximal contractions, 4 MVCs (~ 30 s apart) were performed, each lasting ~ 4 s with real-time visual feedback and verbal encouragement provided; the highest value was taken as MVC force.

### Intramuscular electromyography setup

Following preparation of the skin (shaving, lightly abrading, and cleansing with 70% ethanol), a 25 mm disposable concentric needle electrode (Model S53156; Teca, Hawthorne, NY, USA) was inserted to a depth of 1–2 cm into the mid muscle belly of VL. A common ground electrode was placed over the patella. The iEMG signals were sampled at 40 kHz and bandpass filtered between 10 Hz to 10 kHz. The iEMG and force signals were recorded and displayed in real-time using LabChart8 software (v8.1.13, AD Instruments, UK).

### Isometric contractions and iEMG signal recording

Participants performed a low-intensity voluntary contraction while the needle was positioned to ensure that sharp MUPs were detected [34]. Then, iEMG signals were recorded as participants completed, with real-time visual feedback, 6 × 20 s, 25% MVC voluntary contractions ~ 30 s apart. The needle was repositioned by a combination of rotating 180° and/or withdrawing by 2–5 mm, respectively, between each contraction.

After completion of the brief isometric contractions, a 13 cm cuff, placed around the upper thigh of the right leg, just below the inguinal crease, was inflated to 220 mmHg for 5 min to restrict arterial and venous lower leg BF [35]. Then, the needle was re-inserted at least 0.5 cm from the original insertion site, positioned to detect sharp MUPs, and a 25% MVC isometric contraction was performed for 3 min during BFR (BFR_3min_). The detected iEMG signal was monitored throughout the BFR_3min_ contraction to ensure a stable needle position and recorded during the final 20 s. Following the BFR_3min_ contraction, a 45 s rest was given, but with BFR maintained, then an iEMG signal was recorded during a 20 s, 25%MVC isometric contraction (recovery 1). The cuff was then released, and an additional 45 s rest was given. Finally, an iEMG signal was recorded during a 20 s, 25% MVC contraction.

### Intramuscular electromyographic signal analyses

The procedure for analyzing iEMG signals is described elsewhere [28, 29]. Briefly, using decomposition-based quantitative electromyography [35], MUP trains (MUPTs), extracted from the sampled iEMG signals, were evaluated through visual inspection and suitable trains that had at least 40 MUPs were accepted for data analysis [28, 29]. For each selected MUPT, markers indicating the onset, end, and negative/positive peaks of the MUP template waveform were manually adjusted, where required. MUP duration (ms) was measured as the time between the onset to end markers, and MUP area (*μ*V·ms) was the total area within the MUP duration. MUFR was calculated as the mean rate of consecutive observations of the same MUP, expressed in Hz [36].

### Plasma nitrite (NO_2_^−^) concentration

A 5 mL venous blood sample from an antecubital vein was collected into a lithium-heparin tube (Vacutainer, Becton Dickinson) and centrifuged at 3500 × *g* for 10 min at 4 °C (Hettich^®^ 320 centrifuge, Canada). Plasma was extracted into 1.5 mL microcentrifuge tubes and frozen at − 80 °C for later analysis of the NO_2_^−^ using ozone-based chemiluminescence as previously described [27, 37].

### Statistical analysis

Normality of all data was confirmed using the Shapiro–Wilk test. A paired *t*-test was used to test for differences between NIT and PLA supplements in plasma NO_2_^−^. Two-way repeated measures ANOVA was used to determine *supplementation* × *time* interactions for MUP size (duration and area), and MUFR. Bonferroni corrected paired *t*-tests were used for *post-hoc* paired comparisons when there was a significant main or interaction effect. Cohen’s *d* effect sizes were determined for each paired comparison as: large *d* > 0.8, moderate *d* = 0.8 to 0.5, small *d* = 0.5 to 0.2, and trivial d < 0.2 [38]. Statistical significance was *p* < 0.05 and reported except in cases where *p* ≤ 0.001. Statistical analysis was completed using SPSS 28.0 (IBM Corp., Armonk, NY) and data are presented as mean ± SD.

## Results

### Plasma NO_2_^−^

Plasma NO_2_^−^ concentration was higher following NIT (482 ± 92 nmol·L^−1^) compared with PLA (198 ± 48 nmol·L^−1^) supplementation (*p* < 0.001).

### Neuromuscular responses during contractions

The mean number of MUs sampled per person in the NIT and PLA cohorts respectively were 34 ± 8 vs. 33 ± 8 during the brief isometric contractions, 7 ± 2 vs. 8 ± 1 during the contraction at the end of BFR_3min_, 6 ± 2 vs 7 ± 1 during the contraction at the recovery 1 stage, and 7 ± 3 vs. 8 ± 2 during the contraction at the recovery 2 stage.

**Fig. 2:**
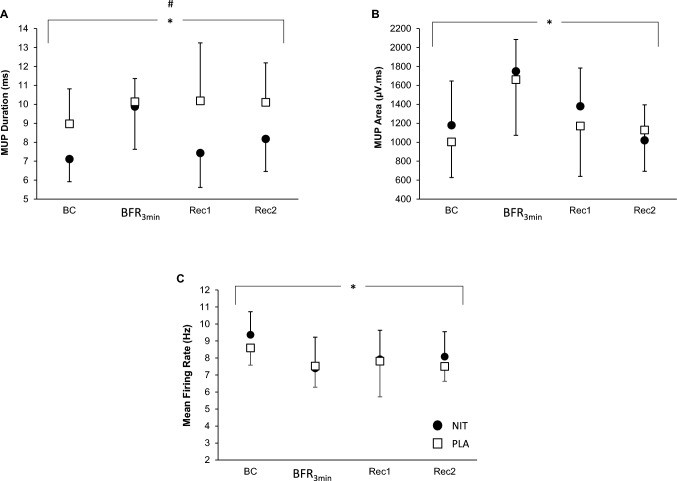
Motor unit potential (MUP) duration (**A**), MUP area (**B**), and MU firing rate (**C**) during the brief isometric contractions (BC), after the BFR_3min_ contraction, and after brief recoveries with (Rec 1) and without (Rec 2) BFR for the nitrate (NIT) and placebo (PLA) cohorts (*n* = 14). Data are mean ± SD. #Main effect of supplement *p* < 0.05. *Main effect of study time point, *p* < 0.05

*MUP Duration* There was an effect of supplement on MUP duration (*F* = 24.05, *p* < 0.001; ŋ_p_^2^ = 0.686, Fig. 2A). *Post-hoc* pair-wise comparisons showed that the mean score for MUP duration was shorter in the NIT cohort than in the PLA cohort for MUs sampled during the brief isometric contractions (7.1 ± 1.3 vs. 9.5 ± 1.8 ms, *p* < 0.001, *d* = 1.6), and at the recovery stages with BFR (7.4 ± 1.9 vs. 10.2 ± 3.1 ms, *p* = 0.002, *d* = 2.8) and without BFR (8.3 ± 1.6 vs. 10.1 ± 2.1 ms, *p* = 0.012, *d* = 2.3). There was also a main effect of the study time point (*F* = 3.34, *p* = 0.039; ŋ_p_^2^ = 0.217); however, there was no interaction between NIT and PLA supplementation and study time point (*F* = 2.46, *p* = 0.090; ŋ_p_^2^ = 0.170).

*MUP Area:* There was no *supplementation* × *time* interaction effect (*F* = 0.642, *p* > 0.05; ŋ_p_^2^ = 0.055, Fig. 2B) nor an effect of supplementation on MUP area. (*F* = 0.002, *p* = 0.968; ŋ_p_^2^ < 0.001) There was an effect of the study time point on MUP area (*F* = 17.90, *p* < 0.001; ŋ_p_^2^ = 0.619). Paired comparisons showed smaller MUP areas during the brief isometric contractions (1158.82 ± 83.23 µV·ms) than after the BFR_3min_ contraction (1709.64 ± 86.81 µV·ms, *p* = 0.002); MUP area after the BFR_3min_ contraction was larger than at recovery 1 (1310.30 ± 89.77 µV·ms, *p* = 0.014) and at recovery 2 (1107.11 ± 80.75 µV·ms, *p* < 0.001).

*MUFR:* There was no *supplementation* × *time* effect (*F* = 0.604, *p* > 0.05; ŋ_p_^2^ = 0.063, Fig. 2C) nor an effect for supplementation on MUFR (*F* = 1.63, *p* > 0.05; ŋ_p_^2^ = 0.153). There was a significant time effect on MUFR (*F* = 7.16, *p* = 0.010; ŋ_p_^2^ = 0.443). *Post-hoc* Pair-wise comparisons show that the mean MUFR was higher during the brief isometric contractions (9.0 ± 0.3 Hz) than after the BFR_3min_ contraction (7.3 ± 0.3 Hz, *p* < 0.001) and at recovery 2 (7.7 ± 0.2 Hz, *p* = 0.003).

## Discussion

The present study aimed to evaluate the effect of NO_3_^−^ supplementation on MU activity following a sustained ischemic contraction, and whether any such effects differ with and without BFR. The principal findings show that 5-days of NO_3_^−^ supplementation, which elevated plasma NO_2_^−^ concentration, shortened MUP duration compared to PLA, but had no effect on MUP area, or MUFR during a sustained ischemic contraction and subsequent brief recoveries with and without BFR. There is a reduction in the mean MUFR during the sustained isometric contraction with BFR and it remained low after brief (~ 45 s) recoveries with and without BFR. These findings provide original data highlighting the potential for NO_3_^−^ supplementation to improve aspects of post exercise neuromuscular recovery, at least following a sustained ischemic isometric contraction.

In the present study, 5-days of NO_3_^−^ supplementation increased plasma NO_2_^−^ concentration by 143% compared with the placebo. This result is in line with previous reports of a comparable increase in plasma NO_2_^−^ after ingesting a similar NO_3_^−^ dose [11, 27, 39, 40]. Improved muscle metabolic recovery following elevation of NO_2_^−^ concentration in plasma has been reported in previous studies [4, 18], however, aspects of neuromuscular function post-exercise are less well-understood.

MUP duration was shorter during brief isometric contractions with NO_3_^−^ supplementation in the NIT cohort compared to the PLA cohort. In addition, while MUP duration increased during the 3 min sustained ischemic contraction with and without NO_3_^−^ supplementation, there was an effect of NO_3_^−^ supplementation on MUP duration after the brief recovery periods, with post hoc analyses showing that increased MUP duration was lowered in only the NIT cohort. In a previous study by McManus et al. [41], MUP duration was shown to return to initial values after a 10 min recovery period, but our data suggests this recovery may be expedited after NO_3_^−^ supplementation. Since restoration of MUP duration after recovery is related to a recovery of muscle fibre conduction velocity (MFCV), the NIT-induced lowering of MUP duration might be due to faster muscle fibre action potential propagation [41–43]. During prolonged muscle contraction, increased accumulation of extracellular potassium (K^+^) is associated with a reduction in muscle excitability and ultimately a reduced MFCV [44–46]. Since Wylie et al. [47] reported a tendency for plasma K^+^ to be reduced during exercise with NO_3_^−^ supplementation, shorter MUP duration in the present study might be related to enhanced K^+^ handling, therefore preserving sarcoplasmic Ca^2+^ release [41, 48]. It is also possible that a reduction in MUP duration might have been caused by a restoration of MFCV and Ca^2+^ release from the SR [49, 50] and hence, likely restoration of contractile force post-recovery [41, 48]. Importantly, in support of this, there is a positive association between sarcoplasmic reticulum Ca^2+^ release and the speed of the action potential propagation along the fibre membrane [48]. Further, NO has been shown to augment neurotransmitter release (e.g., acetylcholine) at the NMJ (12–14) through posttranslational S-nitrosylation of key regulatory protein thiols [15] in rodent models. Given that improved acetylcholine release can enhance motor neuron depolarisation [20], shorter MUP duration might be linked to increased or/and preserved acetylcholine release and subsequently faster MFCV [51]. However, the effect of NO on neurotransmitter release at the NMJ and its elevation through dietary NO_3_^−^ supplementation, remains to be elucidated in humans. Collectively, these findings indicate that NO_3_^−^ supplementation might preserve and/or restore muscle excitability after brief recovery following a prolonged/fatiguing task.

The present data also showed that MUP area increased during a 3 min isometric contraction conducted under ischemic conditions and decreased after brief recovery periods with and without BFR. While these findings are consistent with previous work reporting MU recruitment during fatiguing contractions using intramuscular [42] and surface decomposition techniques [41], the present study is the first to report MUP characteristics during intervening recovery periods following a sustained contraction. The increase in MUP area has been attributed to the recruitment of larger MUs to compensate for the fatigue related reduction in force generating capacity [41]. However, the changes in MUP area following brief periods of recovery were similar with and without NO_3_^−^ supplementation, which are partly in line with our previous observation [11]. This lack of effect might be due to single and low contraction intensity (i.e., 25% MVC). Since changes in MU activity and the efficacy of NO_3_^−^ supplementation in muscle contraction might be task-dependent [32, 52], NO_3_^−^ supplementation might still have potential benefits on MU activity at different intensities (e.g., high) and contraction tasks (e.g., intermittent). Concurrently, there is a reduction in the MUFR at the end of the 3 min ischemic contraction and it remained low after brief periods of recovery with and without BFR and with and without NO_3_^−^ supplementation. These findings are in line with previous reports of a similar pattern in MUFR during sustained isometric prolonged and/or fatiguing contraction [24, 52–54] and are likely due to the increased accumulation of metabolites and subsequent stimulation of muscle afferents [56–58]. Based on some previous reports of beneficial effects of NO_3_^−^ on blood flow, Pi accumulation and PCr recovery under hypoxic conditions [4, 18, 25], the present study hypothesized that NO_3_^−^ supplementation would enhance restoration of MU activity following brief periods of recovery following sustained ischemic exercise by improving physiological and metabolic responses. In contrast to this hypothesis, the present data show that NO_3_^−^ supplementation had no effect on MUFR during the ischemic sustained contraction or after brief periods of recovery with or without BFR. Although ischemia creates a convenient condition to facilitate reduction of NO_2_^−^ to NO [1, 2], the inhibitory effect of ischemia itself, could have been hyper-excitable given the duration of ischemia [59, 60], which is a potential explanation for the unchanged MUFR with NO_3_^−^ supplementation in BFR.

The findings of the present study contrasts with the only previous study that reported increased firing rates from pre- to post across a fatiguing protocol (dynamic box squat: squatting exercise by sitting back on the box) with NO_3_^−^ supplementation compared with placebo, indicating enhanced MUFR after 45 s of recovery [10]. The most obvious explanation for the disparate results between the present and previous study is the task dependency of exercise (dynamic *vs.* isometric exercise) and application of BFR [52] in which changes in MUFR patterns depend on the task being performed. Another possible explanation is that we measured and demonstrated a significant increase in plasma NO_2_^−^ while Flanagan et al. [10] did not. Given that Flanagan et al. [10] administered a NO_3_^−^- rich sport bar that provided a small NO_3_^−^ (~ 0.5 mmol/day) dose, it is possible that other nutrients in the bar (e.g., antioxidants, polyphenols) rather than NO_3_^−^ may have contributed to the effects observed by Flanagan et al. [10].

It is important to highlight that the current study investigated the effect of NO_3_^−^ supplementation on changes in MU activity in response to brief periods of recovery (partial recovery) as an aspect of the neuromuscular recovery process, but the potential effect of NO_3_^−^ supplementation on metabolic recovery cannot be ruled out [4, 18]. Future studies should measure muscle function and metabolic responses in combination with MU activity to improve the understanding of mechanisms by which NO_3_^−^ supplementation may enhance recovery processes after exercise. Given that the present study aimed to investigate the effects of NO_3_^−^ supplementation on MU activity after brief periods of recovery following a sustained ischemic contraction, the influence of NO_3_^−^ on the restoration of MU activity in recovery following completion of other specific relevant tasks, such as intermittent contractions require further investigation.

## Conclusion

In conclusion, the present study shows some alterations in MUP properties in response to brief periods of recovery with and without BFR. Specifically, short-term NO_3_^−^ supplementation, in the form of concentrated beetroot juice, can expedite the recovery of MUP duration following a sustained ischemic contraction in healthy adults. These novel observations improve the understanding of the effects of NO_3_^−^ on post exercise recovery of neuromuscular function, which may have implications for recovery of muscle contractile function and athletic performance. Accordingly, NO_3_^−^ supplementation may have potential as a nutritional ergogenic aid by improving post exercise neuromuscular recovery.

## Data Availability

The data that support the findings of this study are available from the corresponding author upon reasonable request but are not openly available due to privacy or ethical restrictions.
